# MOVING FORWARD TOWARD A NURSING HOME MEASURE FOR GOAL CONCORDANCE AND EVALUATION OF RESIDENT GOALS

**DOI:** 10.1093/geroni/igae098.1216

**Published:** 2024-12-31

**Authors:** Tara McMullen

**Affiliations:** Centers for Medicare & Medicaid Services, Baltimore, Maryland, United States

## Abstract

Longstanding evidence suggests that the goals, preferences, and priorities (GPP) of nursing facility residents may not be adequately addressed during episodes of care. Aligning care with residents’ GPP represents an opportunity to improve outcomes and reduce disparities in the resident population. The 2022 National Academies of Sciences, Engineering, and Medicine (NASEM) report recommended improvement in quality measures under the domain of resident and family experience, along with reporting of these measures on Care Compare to improve shared decision-making and transparency. The subcommittee on quality improvement and measurement is focused primarily on measures of GPP as a useful addition to CMS’ plans to expand the Quality Reporting Program (QRP) measure set. To develop a reliable measure of concordance, this committee explored and assessed the alignment between the individual goals set by residents and the care interventions provided by the staff in routine care delivery. In an evaluation that employed in-depth interviews and reviews of the resident care plan and portions of the medical record, this group of subject matter experts attempted to gain a better understanding of the concordance between resident goals and care provision with the intent to identify areas for improvement in care delivery and enhanced person-centered care practices. In this presentation, committee members will discuss the development and outcomes of this evaluation along with an update on the development of a care concordant measure for nursing home settings.

